# Modifying a Mammography Decision Aid for Older Adult Women with Risk Factors for Low Health Literacy

**DOI:** 10.3928/24748307-20210308-01

**Published:** 2021-04

**Authors:** Tamara Cadet, Gianna Aliberti, Maria Karamourtopoulos, Alicia Jacobson, Morgan Siska, Mara A. Schonberg

## Abstract

**Background:**

Guidelines recommend that before being offered mammography screening, women age 75 years and older be informed of the uncertainty of benefit and potential for harm (e.g., being diagnosed with a breast cancer that would otherwise never have shown up in one's lifetime); however, few older women are informed of the risks of mammography screening and most overestimate its benefits.

**Objective:**

The aim of this study was to learn from women older than age 75 years who have predisposing risk factors for low health literacy (LHL) how they make decisions about mammography screening, whether an existing decision aid (DA) on mammography screening for them was acceptable and helpful, and suggestions for improving the DA.

**Methods:**

We conducted semi-structured interviews with 18 women who were between ages 75 and 89 years and had predisposing risk factors for LHL (i.e., answered somewhat to not at all confident to the health literacy screening question “How confident are you filling out medical forms by yourself?” and/or had an education level of some college or less).

**Key Results:**

Findings indicate that women in this study lacked knowledge and understanding that one can decide on mammography screening based on their personal values. Women were enthusiastic about screening based on an interest in taking care of themselves but rely on their providers for health care decisions. Overall, most women found the DA helpful and would recommend the use of the DA.

**Conclusions:**

Findings from this study provide formative data to test the efficacy of the modified DA in practice. Failing to consider the informational needs of adults with LHL in design of DAs could inadvertently exacerbate existing inequalities in health. It is essential that DAs consider older women's diverse backgrounds and educational levels to support their decision-making. **[*HLRP: Health Literacy Research and Practice*. 2021;5(2):e78–e90.]**

**Plain Language Summary:**

The goal of this research was to understand how women older than age 75 years with risk factors for low health literacy made decisions about getting mammograms, whether an educational pamphlet was helpful, and suggestions for improving it. This research helps in understanding how to involve this population in the process of designing patient-related materials for mammogram decision-making.

Women age 75 years and older are the fastest growing segment of the population in the United States. Even though breast cancer incidence increases with age (American Cancer Society, 2017; Roberts et al., 2018), none of the randomized trials of mammography screening included this age group, and it is not known if mammography helps these women live longer (Elomrani et al., 2015; Walter & Schonberg, 2014). The benefits of mammography are uncertain, particularly for older women with short life expectancies, and there are important harms to screening including pain and anxiety related to the test, complications from additional tests (e.g., breast biopsy) after a false-positive mammogram, and overdiagnosis of tumors that are not life-threatening. Overdiagnosis is particularly concerning because risks, such as pain, infections and swelling in their arm after mastectomy, bone pain, and osteoporosis after taking hormonal pills (Ramin et al., 2018) of breast cancer treatment increase with age (Barratt, 2015; Welch et al., 2016). Guidelines state that there is insufficient evidence to recommend mammography screening for women older than age 75 years and recommend that older women be informed of the uncertainty of benefit and the potential for harm. Guidelines further encourage clinicians to consider patient health and life expectancy before offering screening (Oeffinger et al., 2015; U.S. Preventive Services Task Force, 2019). However, Medicare covers annual mammograms for all women older than age 65 years and many older women are screened regardless of their life expectancy (Medicare, 2019). Screening among women in the U.S. is opportunistic regardless of age; women may ask for a mammogram or a clinician may recommend mammography. In contrast, other countries, including most European countries, use population-based screening where women age 50 to 69 years are invited to be screened approximately every 2 years through governmental programs (International Agency for Research on Cancer, 2016).

Often, older adult women are not informed of mammography harms and most overestimate the benefits (Walter & Schonberg, 2014). To help this population of women better understand both the benefits and risks of a mammography screening to inform their decision-making, a decision aid (DA) was developed (https://eprognosis.ucsf.edu/decision_aids/Mammography_75-84.pdf). That DA, however, was not developed or tested considering the specific needs of older women with low health literacy (LHL) (Schonberg et al., 2014). Another DA on mammography screening was developed in Australia (Ottawa Hospital Research Institute, 2019) to help women age 70 years decide whether to continue screening. However, because screening is widely recommended until age 74 years in the U.S. and because this present study was U.S. based, we aimed to improve the health literacy components of the mammography screening DA previously developed from UCSF for women older than age 75 years.

## Mammography Decision Aid

DAs are educational tools used to inform patients about available treatments and their potential benefits and risks. DAs have been shown to increase patient knowledge of the benefits and risks of treatment, increase patient participation in decision-making, and improve decision quality (Stacey et al., 2017; van Weert et al., 2016). The mammography DA previously developed is paper-based and includes information on older adult women's breast cancer risk, life expectancy, outcomes of screening, competing mortality risks, breast cancer treatments, and a values clarification exercise.

In a pilot pretest/posttest study of that DA, which included 45 women older than age 75 years, participants increased their knowledgeable about the benefits and risks of mammography and, as a result, fewer women with short life expectancies intended to be screened. Educational attainment among the participants was the following: 4 (9%) had less than a high-school education, 14 (31%) had a high-school education, and 27 (12%) had completed some college education. Although women with a high-school education or less found the DA acceptable, they were less likely to have improved knowledge of the benefits and risks of mammography screening after reading the DA and were more likely to report that the DA made them feel anxious compared to women with higher education: 44% (*n* = 8) vs. 19% (*n* = 5; *p* = .07). Also, women with less than a high-school education needed more support from the research assistant to use the DA (Schonberg et al., 2014).

### Importance of Considering Health Literacy

Health literacy is “the ability to obtain, process, or understand basic health information needed to make appropriate health care decisions” (U.S. Department of Health and Human Services, 2010). LHL is associated with low educational attainment, adults older than age 75 years, racial and ethnic identity, and low income (U.S. Department of Health and Human Services. Office of Disease Prevention and Health Promotion, n.d.). LHL is also associated with a lack of medical knowledge, low self-efficacy, and less desire to participate in treatment decision-making (Findley, 2015). Furthermore, the physiological and psychological changes of aging along with social and cognitive issues that may come with aging can affect literacy levels of older adults (Kobayashi et al., 2015).

A systematic review (McCaffery et al., 2013) found that few existing DAs across health conditions address the needs of populations with LHL, and the specific effects of DAs designed to diminish the influence of LHL are not known. Further, they concluded that DAs need to be developed that are appropriate for all health literacy levels. Previous research also suggests that although patients with LHL are less likely to want to engage in decision-making, they are also not aware of their options to participate in decision-making and may want to be engaged in the decision-making process (Politi et al., 2013; Seo et al., 2016). Therefore, interventions are needed to help patients with LHL to participate in decision-making.

Furthermore, mammography screening DAs for populations with LHL have been targeted for women in their 40s (Eden et al., 2015; Elkin et al., 2017). Given the increasing population of women who are older than age 75 years, there is a need for mammography DAs designed for older adult women with LHL to inform them of the uncertainty of the harms and benefits associated with mammography screening. Thus, this study will add to the knowledge base by sharing findings from semi-structured interviews to modify a DA on mammography screening for older adult women with LHL.

### Objective

National recommendations suggest that health educational materials be written using low literacy principles so that all patients can benefit from these tools (Hersh et al., 2015; Muscat et al., 2015). Given appropriate support, older adults of all literacy levels can participate in medical decisions (Krist et al., 2017). Despite this, few DAs are written for patients with LHL and further research is needed to ensure that these populations have access to tools to support their decision-making (Koops van't Jagt et al., 2016; McCaffery et al., 2013). Thus, the purpose of this study was to understand how women make decisions about mammography screening and to modify the existing mammography DA for women older than age 75 years for use among older adult women with LHL. Specifically, the research question that guided the interviews was: “How can we design a mammography decision aid that is understandable for older women with low health literacy?” The DA was evaluated on its acceptability for older adult women with LHL.

### Conceptual Framework

The Integrative Model of Behavior Prediction (IMBP) is a framework that posits a reasoned action to understanding behaviors, suggesting that there are many variables that may influence behaviors in some ways but only a small number of variables are needed to change a particular behavior in a particular population (Fishbein & Ajzen, 2010). Specifically, IMPB suggests that intention to perform a behavior originates from specific beliefs that people have about the behavior. The beliefs may be reasonable but may not be rational. In addition, if people believe that performing or participating in a specific behavior is a good thing, they are more likely to be motivated to actually perform or participate in the behavior than if they believe performing or participating the behavior is a bad thing. Thus, IMPB can account for any behavior regardless of rationality.

Further, IMPB suggests there are five determinants that directly affect behavior. The most important determinant is intention for a decision. First, behavioral intention is determined by attitude, perceived norms, and personal agency (self-efficacy/perceived power). Second, a person needs the knowledge and skills to carry out the behavior. Third, the behavior should be relevant to the person. Fourth, there should be few or no environmental constraints that make behavioral performance difficult. Finally, with experience performing the behavior, the behavior will become habitual for the person.

Attitude is a person's evaluation of how favorable or unfavorable performing a particular behavior would be and can be considered as experiential attitudes (feelings about the behavior) and instrumental attitudes (behavioral beliefs). Perceived norm is the social pressure a person expects regarding a behavior. There are two types of norms that make up perceived norm: injunctive (normative beliefs/other's expectations) and descriptive (normative beliefs/other behaviors). Personal agency is a person's capability to originate and direct actions for given purposes. Specifically, self-efficacy and perceived control make up personal agency. Self-efficacy is a person's capability to originate and direct actions for given purposes. Perceived control is a person's perception of the degree to which various environmental factors make it easy or difficult to perform a behavior.

**Figure 1.**
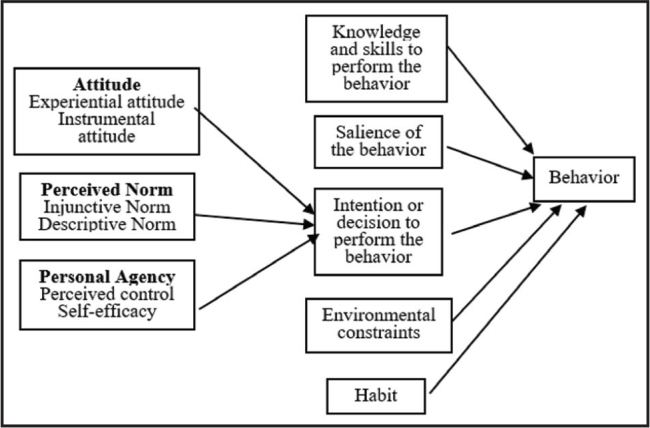
Conceptual framework for the overall study and analyses of the interviews.

Empirically based literature has used IMPB as the theoretical framework in the design and development of DAs to better understand the effects of the DAs on decision-making. Specifically, Frosch et al. (2008) suggest that DAs may help to influence the beliefs, underlying attitudes, social norms, and self-efficacy because DAs often highlight the role of the patient's preferences when making a decision, possibly resulting in more positive attitudes about taking an active role in decision-making. Further, Frosch et al. (2009) note that formative qualitative research is necessary to draw out beliefs underlying the key constructs and help target the DA to increase the likelihood that the DA may influence decision-making. In addition, they note that investigations focused on the development of DAs can consider the role that determinants of screening participation play as part of the design using constructs of IMPB. Thus, it is important to understand the role that the determinants of mammography screening play as part of the design-using constructs of IMPB (**Figure 1**).

## Methods

### Study Design

Using semi-structured interviews, we aimed to modify an existing mammography screening DA for women older than age 75 years with predisposing risk factors for LHL. We recruited women for this study from five sites including a large Boston-based academic medical center, a satellite primary health clinic, one community health center affiliated with the medical center, and two community-based organizations. The community clinics and health centers predominantly serve patients from lower socioeconomic backgrounds. Inclusion criteria for all women regardless of sites included women between ages 75 and 89 years, women who had some college or less educational attainment, and women who were considered to have LHL due to the response of *somewhat to not at all confident* to the validated health literacy question “How confident are you filling out medical forms by yourself?”

We initially considered identifying women with predisposing risk factors for LHL based solely on the response to the question, “How confident are you filling out medical forms by yourself?” However, we learned that older adult women felt reluctant to admit their discomfort with medical forms. Using this question as our only condition for defining a woman's literacy level led us to initially exclude women for whom the DA may have been useful. Therefore, we added educational attainment for all women to our criteria for defining older adult women's health literacy. Consistent with the literature (Easton et al., 2013), we found that there was a stigma about being considered to have risk factors for LHL and participants were concerned about responding to this question. One consequence of LHL is the potential of people feeling that there was a stigma about being considered “at risk” for LHL and being perceived as having low intelligence (Mackert et al., 2011). Thus, it may be difficult for people to share concerns about understanding and using health information (Batterham et al., 2016). Furthermore, to ensure that we were reaching the intended population, educational level was added based on research about the intersectionality that exists between educational attainment, age, racial and ethnic identity, socioeconomic status, and LHL (Flores & Halsall, 2017; van der Heide et al., 2013; Wolf et al., 2010). Given the intersection of these factors, it was important to use as many of those factors as possible to assess health literacy.

***Exclusion criteria.*** Women older than age 90 years were excluded because, on average, they have less than a 5-year life expectancy (Arias, 2012), may have dementia, which is common (36%) among this population and few are screened (Plassman et al., 2007); they have a history of breast cancer; their medical records indicated no history of a mammogram in the past 3 years; and they scored >9 on the Orientation-Memory-Concentration Test (Katzman et al., 1983), indicating cognitive impairment. We also excluded women without the capacity to participate. Women were asked seven questions about what participating in the study entailed and women were only included if they answered four or more questions correctly.

## Participants

We identified participants via electronic medical records (EMR) and through community-based organizations' contacts. To complement identifying participants at the academic primary care clinic, we also accessed EMR at a satellite clinic and at a community health center affiliated with the academic primary care clinic according to our exclusion criteria. For women identified through EMR, we first obtained approval from their primary care physicians (PCPs). After receiving approval from PCPs, we mailed eligible patients an informational letter about the study and a number to call if they wanted to opt-out of being contacted. For women identified through community-based organizations, we assessed eligibility before proceeding to ask them to participate. We met all women who agreed to be interviewed at a time and place convenient to them (often their homes) and obtained written informed consent. We conducted reminder calls a week before and the day before the interview. Participants were offered $40 for participating in the study.

### Interview Structure

We used an in-depth semi-structured interview to understand the needs, interests, and behaviors of participants and used cognitive interviewing to get feedback on the DA. The interviews were conducted by two investigators (A.J. and T.C.) with training in qualitative research. A.J. and T.C. conducted three interviews together; A.J. conducted 12 alone and T.C. conducted 3 alone. In-depth interviews are useful in gaining insight into participants' beliefs, knowledge, and experiences (Padgett, 2016). We adapted questions previously used to evaluate the DA for older adult women to understand participants' knowledge of mammograms, their decisions to have or not to have a mammogram, and factors that have influenced their decisions. We then asked participants to review the DA in detail, going over each line, and to “think-aloud” about the content. We asked participants to say everything that passed through their mind as they read. Cognitive interviews using this think-aloud approach allow researchers to understand how patients perceive and interpret the information presented and to identify any potential problems in the material (Padilla & Leighton, 2017). We asked participants about what they found confusing and what they did and did not understand about the existing DA. In addition, we asked about the DA's acceptability—the length, clarity, whether they found the materials anxiety invoking, and/or whether they would recommend the materials to a friend. At the conclusion of the interview, participants were asked about their demographics, health status, and functional capacity. Each interview took approximately 1 hour.

According to Rudd (2014), formative research and pilot testing are among the recommended strategies to examine the language, organization, and structure of materials such as DAs in collaboration with and feedback from members of the intended audience. Two components of obtaining feedback include asking questions of the intended audience and applying the Teach-Back method (i.e., having the patient repeat key information). We incorporated these health literacy principles as part of the interviews.

### Data Analysis

All interviews were audio-taped and transcribed verbatim using an external professional transcription service. Copies of all transcripts were distributed to each member of the analysis team (T.C., M.S., and M.A.S.). Qualitative analyses were conducted using an iterative process and following standard techniques (Miles et al., 2014). Two members (T.C. and M.A.S.) reviewed the first four transcripts line by line in their entirety to identify comments related to the IMBP conceptual framework. As a means of triangulation (Padgett, 2016), T.C. and M.A.S. met two times to identify a total of 10 codes that reflected the conceptual framework. Furthermore, T.C. and M.S., the primary coders, independently reviewed, made observations, and coded each transcript, then met to review their findings. The primary coders first manually coded the transcripts, employing a provisional code list based on our theoretical framework first developed by T.C. and M.A.S. in their review of the first four transcripts. The provisional codes were refined and revised throughout the coding process by the research team. These first order codes were assigned operational definitions (Miles et al., 2014; Padgett, 2016). Using observer triangulation, one team member (M.S.) recoded the transcripts using the predetermined codes (Miles et al., 2014; Padgett, 2016) using NVivo 12 qualitative data analysis software (QSR International, 2018). To ensure trustworthiness in the coding process, the second team member (T.C.) then reviewed coding (Padgett, 2016). Discrepancies in coding were resolved through consensus among each of the two team members and the lead investigator. To further ensure credibility, the lead investigator reviewed every third manuscript. The second order coding occurred as we reviewed the codes to identify semantic relationships in our findings, which were used to group them into two overall themes: patient factors and perceived norms.

The DA was revised between every two and three interviews after listening to the audiotapes and reviewing notes where women expressed confusion with statements, such as “I do not know what you are saying,” “I don't know how to answer that,” or “I don't know what that means, or why is that important.” The team would discuss what the confusion seemed to indicate, and based on health literacy principles and research, we collectively provided suggestions to revise the DA. We kept interviewing women and getting feedback until no new concerns or issues were identified (Francis et al., 2010; Legard et al., 2003; Saunders et al., 2018).

When interpretation of interviews revealed no new significant insights (thematic saturation), we stopped interviewing and discontinued revising the DA. In qualitative research, sampling until the point of redundancy or thematic saturation provides evidence of the credibility of developed theory and is an accepted point to stop sampling subjects. Descriptive characteristics of the sample were determined using STATA Statistical Software (Version 15). Some quotes were edited for grammar. The Institutional Review Board at Beth Israel Deaconess Medical Center approved this study and its ethics.

## Results

Overall, 18 of 50 (36%) of the eligible women approached participated in the study. Of the nonparticipants, 30 women were not interested, and 2 women were interested but were not interviewed. We interviewed until thematic saturation was reached, which occurred at 18 participants. The mean age of the participants was 81 years (range, 75–89), and 6 (33%) were African American (**Table 1**). Most of the women (*n* = 15, 84%) indicated that the length of the DA was just right; most saying that the information was clear and that they would recommend the DA (**Table 2**). Because we incorporated the recognized health literacy principles when interviewing the participants, the themes identified account for the role of literacy.

**Table 1:**
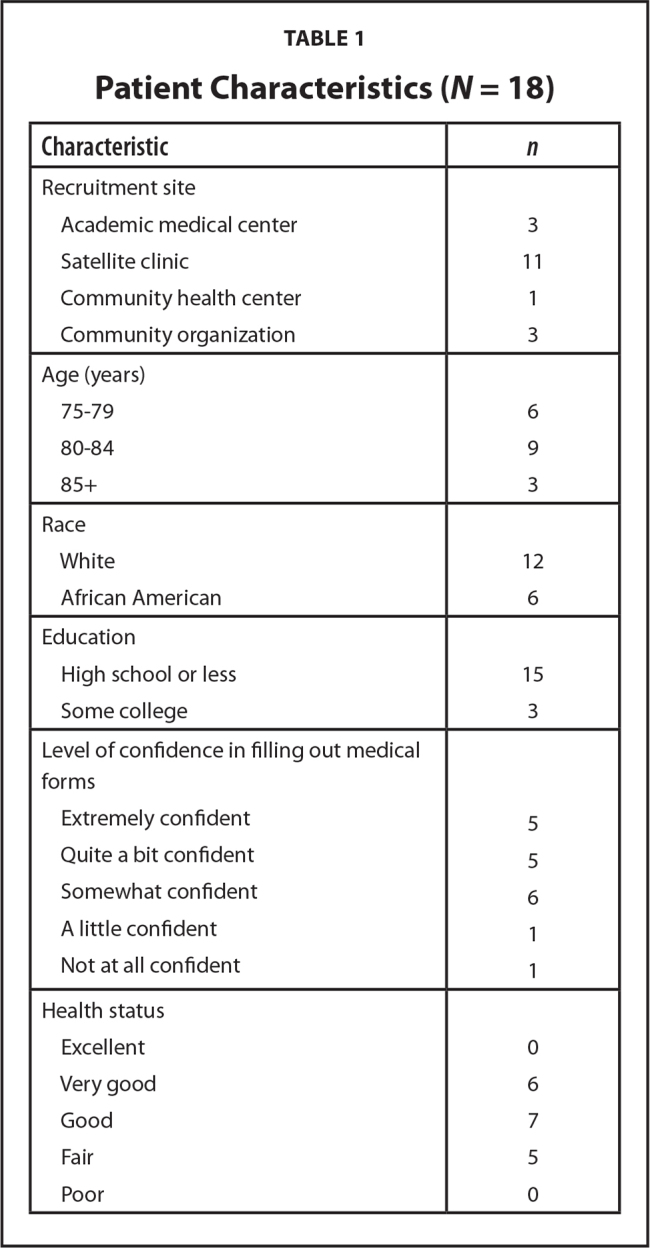
Patient Characteristics (*N* = 18)

**Characteristic**	***n***

Recruitment site	
Academic medical center	3
Satellite clinic	11
Community health center	1
Community organization	3

Age (years)	
75–79	6
80–84	9
85+	3

Race	
White	12
African American	6

Education	
High school or less	15
Some college	3

Level of confidence in filling out medical forms	
Extremely confident	5
Quite a bit confident	5
Somewhat confident	6
A little confident	1
Not at all confident	1

Health status	
Excellent	0
Very good	6
Good	7
Fair	5
Poor	0

**Table 2:**
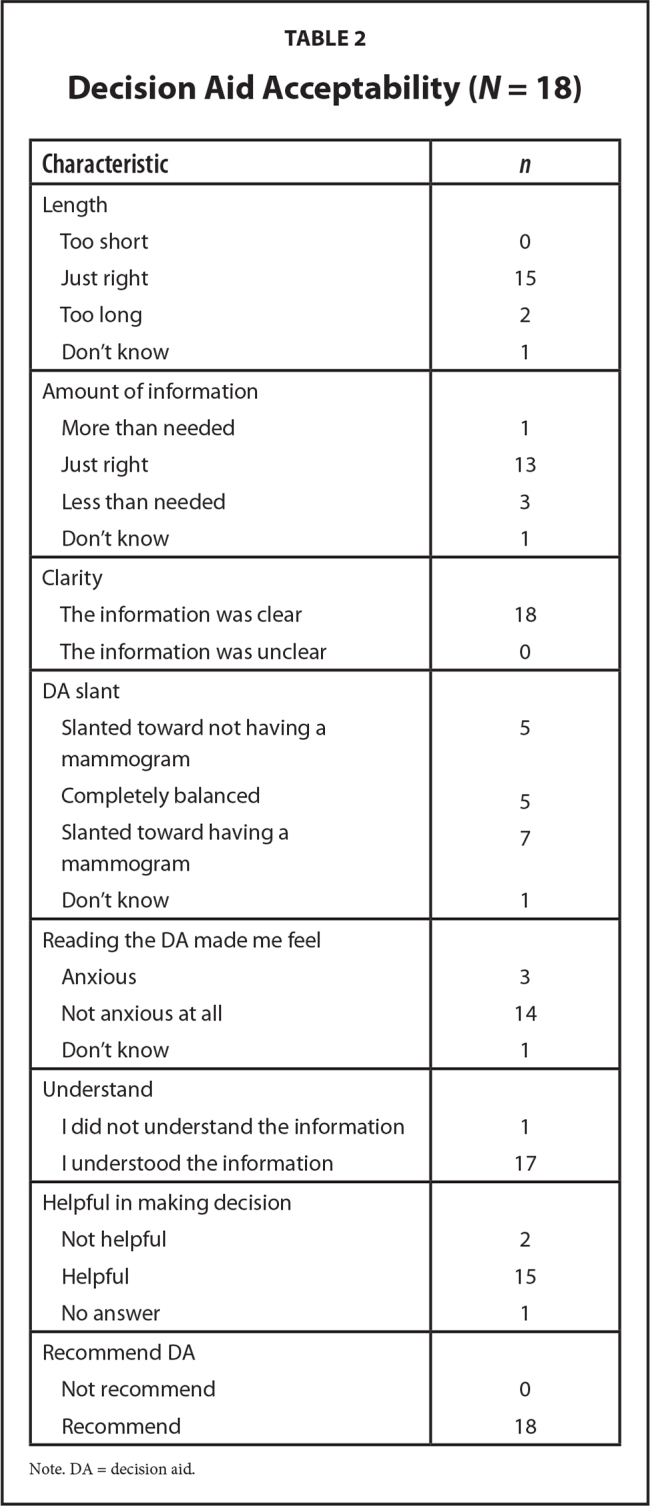
Decision Aid Acceptability (*N* = 18)

**Characteristic**	***n***

Length	
Too short	0
Just right	15
Too long	2
Don't know	1

Amount of information	
More than needed	1
Just right	13
Less than needed	3
Don't know	1

Clarity	
The information was clear	18
The information was unclear	0

DA slant	
Slanted toward not having a mammogram	5
Completely balanced	5
Slanted toward having a mammogram	7
Don't know	1

Reading the DA made me feel	
Anxious	3
Not anxious at all	14
Don't know	1

Understand	
I did not understand the information	1
I understood the information	17

Helpful in making decision	
Not helpful	2
Helpful	15
No answer	1

Recommend DA	
Not recommend	0
Recommend	18

Note. DA = decision aid.

### Themes

Based on the conceptual framework IMBP, we identified two content areas: patient factors and perceived norms. **Table 3** presents each theme and the frequencies of each theme. **Figure 1** illustrates the relationship of these content areas within the conceptual framework. Within patient factors were the following themes: knowledge, self-efficacy, age, and attitudes toward screening.

**Table 3:**
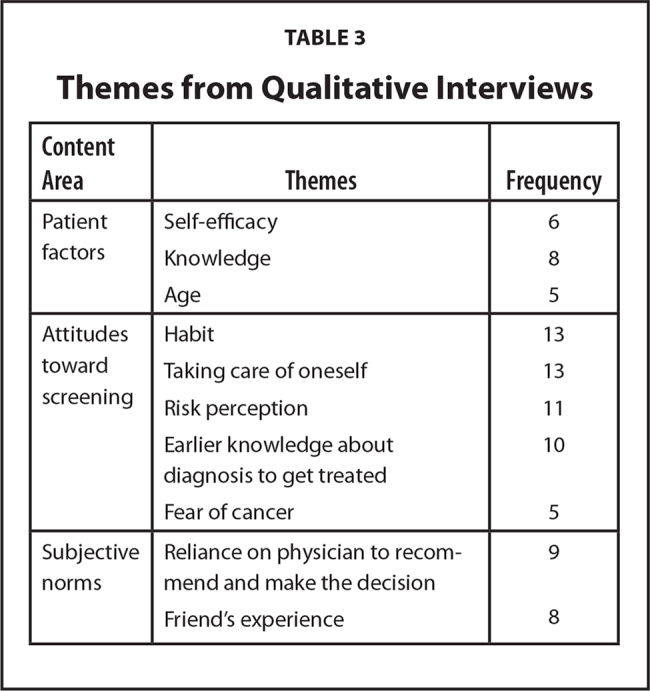
Themes from Qualitative Interviews

**Content Area**	**Themes**	**Frequency**

Patient factors	Self-efficacy	6
	Knowledge	8
	Age	5

Attitudes toward screening	Habit	13
	Taking care of oneself	13
	Risk perception	11
	Earlier knowledge about diagnosis to get treated	10
	Fear of cancer	5

Subjective norms	Reliance on physician to recommend and make the decision	9
	Friend's experience	8

***Knowledge.*** There was a general lack of knowledge about the facts about breast cancer (e.g., breast cancer grows slower in older women or that heart disease kills women more than breast cancer). Part of the lack of knowledge included not believing the facts:

- “I didn't know as you get older, you don't have to have mammograms”
- “. . .but don't say the chance of getting breast cancer goes up with age, because that is not true”

This lack of knowledge seemed to affect women's self-efficacy or confidence to make a decision about discontinuing screening even if their health and life expectancy suggested that they could stop. In other words, participants were not sure about discontinuing screening:

- “Maybe the. . . you know, having a mammogram may increase the number of tests and treatments that I get, and I might not need some of those”
- “So, it's starting to make me wonder if I should continue to have them”

Although a lack of knowledge and self-efficacy to make a decision may contribute to the participants' perspectives, there were some women who recognized that age was an important consideration in deciding whether to continue or discontinue mammography screening:

- “At my age—I don't need to worry about it. The nice thing, after you get a certain age, you don't feel that you need it”
- “I personally believe when you get a certain age, you do stop worrying about mammograms and I don't think many people do”

Whereas some women felt age was an important consideration, others reflected their important factors based on their attitudes toward screening. Attitudes toward screening included habit, taking care of oneself, risk perception, earlier knowledge about diagnosis to get treated, and fear of cancer.

Women discussed getting screened for breast cancer as a habit, as something they had to do and just did without thinking much more about it:

- “Well, I believe in it. I believe in it fully. I am going to be 80 as well this year and I've been getting them I can't even remember how many years they go back, and I believe that you should have it anyway”
- “Well, we get them because we want to find out”

In some ways, the women believed that getting a mammogram was part of being healthy—a good habit to have—and were reassured when the results came back normal:

- “Having a mammogram may help me feel good about myself and my health”
- “Because when I get the letter saying your mammogram was good and all that, it's like I take a deep breath and I always say thank you, God. So, I do derive comfort”

Further, participants were clear that family history increased breast cancer risk, but they were less clear about their absolute risk of breast cancer. They did not believe they were affected by the absolute risks:

- “I have to have it (mammogram) because of all the cancer I have in my family”
- “That's somebody's prediction, summary of things. That's not true. That's not true. Two more women out of 1,000 who do not have mammograms diagnosed with breast cancer that had spread outside the breast. I don't believe that either (referring to the absolute risks)”

Finally, women in the study expressed comments about wanting earlier knowledge about diagnosis to get treated, which may be related to the fear of cancer. Collectively and individually, the need to know about a possible diagnosis and the fear of cancer may contribute to the need for screening to alleviate some of the fear:

- “I say, do the mammogram, they can help you, they catch in time before you get the lump thing”
- “Think it's better to find out and know then wait for it to happen. I would rather know. Even if it doesn't cause problems, just to know it's there. I would rather know that I have it”
- “Well, I think it's no good—any kind of cancer no good. You're supposed to treat. You get it maybe everything will be okay”
- “[It] might grow slower, but it's still painful—cancer. And it's scary. I think it's scary more than anything. I think it's better to find out and know instead of just waiting for it to happen”

As discussed earlier, there are two types of norms that make up perceived norms: injunctive (normative beliefs/other's expectations) and descriptive (normative beliefs/other behaviors). Women indicated a reliance on the doctor to recommend and make the decision as well as their friend's experiences. Their perceived norms were mixed. Some indicated that they were not influenced by their friends, whereas some discussed the idea that because other people do it, so should they:

- “I'd ask him questions about what to do”
- “Well, the doctor say to me, I supposed to do, yeah”
- “Most of my friends, I only have a couple left. I would listen to them, but I don't think they'll sway me one way or another”
- “Almost all the ladies I talk to have the mammogram”

This is relevant given the conceptual framework, which suggests that norms can influence a decision (Montano & Kasprzyk, 2015). Specifically, the women's reliance on their doctors to recommend a mammography screening may be related to what they believe doctor think they should do and their motivation to comply. In addition, their friend's experiences also seem to influence their decisions. This is related to descriptive norms, which refers to their perceptions of what others in their social/personal networks are doing.

### Decision Aid Changes

Using the health literacy principle recommended strategy of conducting formative research to examine the language, organization, and structure of materials such as DAs in collaboration with and feedback from members of the intended audience (Rudd, 2014), the DA was iteratively revised as we interviewed women. Using the think-aloud method to get feedback from the women about the DA, the women noted the following problems: the language was too advanced; they had difficulty understanding how to interpret the pictographs; there were not pictures that represented their racial and ethnic backgrounds; there were too many words on a page; they did not understand the tables; they had difficulty understanding what continuing or discontinuing mammograms meant; they had difficulty understanding what it meant to discuss their thoughts with doctors; and that the length was too long. As a result of the feedback, the following changes were made to the DA.

The women found the DA helpful, but some of the information, such as language and pictographs, were confusing. Thus, we eliminated the pictographs and converted the information to text in a table. We created more white space for information making it easier to read. We made three major word changes to address the confusion around risk and overdiagnosis. We added more pictures that represented White and African American women and some of the African American women commented that they appreciated the representation in the pictures. Women were confused about what stopping mammograms meant and so we added explanations about their ability to choose continuing or discontinuing mammograms. We also clarified that they should have discussions about their thoughts with a doctor. Finally, we simplified the phrases about risk factors, decision-making, and pros and cons of mammograms. As a result of the changes, we shortened the DA from 11 to 8 pages (**Table A)**.

**Table A:**
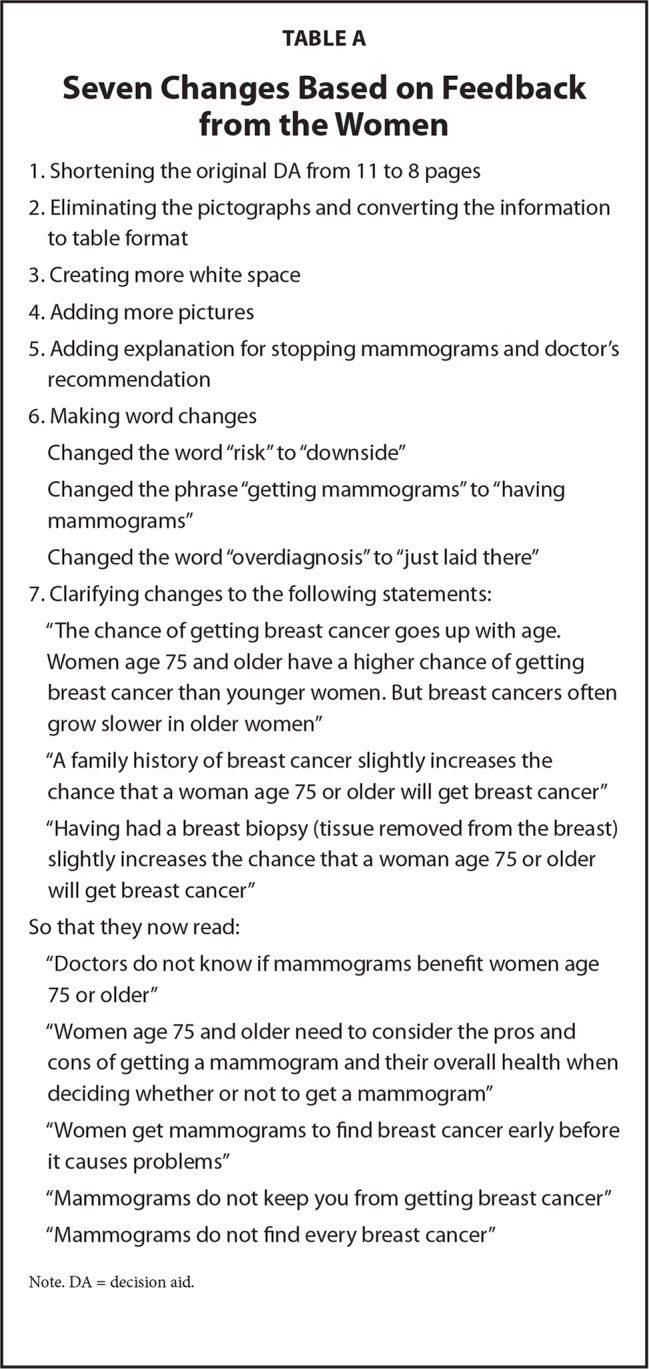
Seven Changes Based on Feedback from the Women

1. Shortening the original DA from 11 to 8 pages
2. Eliminating the pictographs and converting the information to table format
3. Creating more white space
4. Adding more pictures
5. Adding explanation for stopping mammograms and doctor's recommendation
6. Making word changes
Changed the word “risk” to “downside”
Changed the phrase “getting mammograms” to “having mammograms”
Changed the word “overdiagnosis” to “just laid there”
7. Clarifying changes to the following statements:
“The chance of getting breast cancer goes up with age.
Women age 75 and older have a higher chance of getting breast cancer than younger women. But breast cancers often grow slower in older women”
“A family history of breast cancer slightly increases the chance that a woman age 75 or older will get breast cancer”
“Having had a breast biopsy (tissue removed from the breast) slightly increases the chance that a woman age 75 or older will get breast cancer”
So that they now read:
“Doctors do not know if mammograms benefit women age 75 or older”
“Women age 75 and older need to consider the pros and cons of getting a mammogram and their overall health when deciding whether or not to get a mammogram”
“Women get mammograms to find breast cancer early before it causes problems”
“Mammograms do not keep you from getting breast cancer”
“Mammograms do not find every breast cancer”

Note. DA = decision aid.

## Conclusions

Our study explores the perceptions and determinants of women older than age 75 years with predisposing risk factors for LHL and their understanding of a mammography DA designed to provide older adult women with information about the pros and cons of the decision to continue or discontinue mammography screening. We identified themes that influenced women's decisions to continue screening or discontinue screening using the IMBP conceptual framework. The specific themes include patient factors, such as efficacy, knowledge, age, habit, taking care of oneself, risk perception, earlier knowledge about diagnosis to get treated, and fear of cancer; and subjective norms, such as reliance on a doctor to recommend and making a decision based on friends' experiences. Although these themes are recognized by previous investigations, which suggest patient factors and subjective norms are important considerations regarding decision-making among older adult women and mammography (Schonberg et al., 2006, 2007), we discuss these themes given the incorporation of the health literacy principles discussed previously as part of the interviews. We used these themes to help modify the mammography DA in addition to the specific suggestions that women made to better support their decision-making process.

Overall, results suggest that participants lacked the general knowledge about mammograms and the knowledge that one could make a screening decision and can make a decision based on personal values. We made sure the modified DA clearly informed women that it was a personal decision to continue or discontinue mammography screenings. This finding is consistent with Politi et al. (2013), which indicates that patients with LHL were not aware of their options to participate in decision-making. Furthermore, previous research (Durand et al., 2014) indicates that the ability to engage in decision-making requires self-efficacy and the women in this study appeared to lack self-efficacy to make a decision. Perhaps, in part, because they did not know they could make a decision. In addition, it may be that older adults prefer a paternalistic model and it is a cultural, age-expected response to people in authority (Fiks & Jimenez, 2010). Although evidence indicates that adults with LHL tend to be less likely to participate in preventive health services (Fernandez et al., 2016; MacLeod et al., 2017), findings may not be similar for this study. The women in this sample indicated that getting screened was not only a habit but part of taking care of themselves. This, in addition to the need for earlier knowledge about a possible diagnosis so they can get treated, seemed to bias the women toward screening. The need for early detection speaks to the public health message of getting screened regardless of health literacy (Hersch et al., 2017; Schonberg et al., 2013; Torke et al., 2013). Findings (Schonberg et al., 2013) showed no differences in receipt of mammography screening by race in women older than age 75 years, further reinforcing the fact that the public health messages have been successful among all women. Yet, with the shift toward shared decision-making for women older than age 75 years, there is a need to engage women of all literacy levels to make these decisions and tools such as the one we have developed to help them weigh the benefits and risks of mammography screening.

Although getting screened is a habit among older adult women, many women in this study, like other women, are inclined to follow their doctor's recommendation. Findings from Schonberg et al. (2013) indicate that a physician recommendation was the strongest predictor of screening regardless of life expectancy. When we consider women with risk factors for LHL, Morris et al. (2013) suggests that there is a higher likelihood for people with LHL to rely on their physicians compared to those with higher health literacy. Possible reasons for this include the need for assistance in navigating the health care system and understanding what is going on with them. In addition, previous literature may consider this reliance on the doctor as a form of power distance. Power distance is the interpersonal authority or influence that exists between two people. Specifically, in some populations, providers are expected to be the authority or expert who directs their patients' health behaviors by explaining their recommendations (Alden et al., 2015). However, Schoenborn et al. (2019) suggest that physicians were concerned about conversations regarding discontinuing screening. Furthermore, patients report not having a discussion with their physician about discontinuing screening and if a discussion was had, the conversations were brief and limited (Torke et al., 2013).

When considering the role of friends' experiences, the women in our study had mixed experiences. Evidence indicates subjective norms, including that friends do play a role in medical decision-making. Specifically, Brabers et al. (2016) suggested that people are likely to make decisions that others think they should make. Evidence indicates subjective norms, including friends, do play a role in medical decision-making. In contrast, Fransen et al. (2018) noted that there was not a relationship in the role of friends and intentions to make a decision. Perhaps, this finding is explained because the women in the study were more interested in the doctor's opinion than their friends' experience or perhaps the women valued both the doctor's opinion as well as their friends' experiences.

Therefore, having a low literacy DA may help older women prepare for a conversation, particularly because 83% found the DA helpful and 100% would recommend the use of the modified low health literacy DA. Recommending the use of the DA suggests that the women in this study are interested in learning and understanding about making mammography screening decisions. Furthermore, having women feel prepared to have a conversation with their providers may alleviate some of the fears that providers may have about these conversations with older adult women regarding continuing or discontinuing cancer screening (Schoenborn et al., 2019).

Although women were inclined to follow the doctor's recommendation, there were some who would disagree with their doctors if they recommended discontinuing screening. In fact, there were some women who challenged the knowledge in the DA, particularly that there are breast cancers found by mammography that may otherwise not have caused problems in a woman's lifetime. They did not believe the information presented in the DA was true or they were suspicious about the facts that did not specifically apply them. Specifically, findings from Torke et al. (2013) indicate negative responses to recommendations from government panels or population-based statistical information that may not be relevant to a person. In fact, some women in the study indicated that no one could predict what can and cannot happen to an average woman because there is no average experience. These perceptions are also consistent with evidence that suggests that women older than age 70 years lack an understanding about “overdiagnosis” and were suspicious and challenged by the use of the term (Pappadis et al., 2018).

## Study Limitations

There are several limitations to this study including the conditions by which we define risk factors for health literacy. It is increasingly recognized that identifying participants with LHL is a multidimensional construct and we started with one criterion and later added education as an additional criterion. This change in criteria halfway through the study may have introduced differing responses to the DA. Despite the limitations, the strength of this study is a modified version of a mammography DA that has the potential to be useful to all older adult women regardless of their health literacy level because we made the DA shorter, removed pictographs, used simpler language, and clarified that having a mammogram is a decision. Future steps include testing the effect of the DA in a pre-post pilot trial.

Findings from this study provide formative data to test the efficacy of the modified DA in practice. Failing to account for health literacy in design of DAs could inadvertently exacerbate existing inequalities in health (Hasnain-Wynia & Wolf, 2010). It is critical to follow the health literacy principles that suggest collaboration with the target population by getting feedback on materials so that we are not unintentionally excluding their voices. However, we learned, as evident from our response rate, that this population is not accustomed to participating in research studies. Thus, to get participation requires time to explain what it means to be involved in a research study and the commitment required to use qualitative methods. In addition, because evidence (Knighton et al., 2017) indicates that LHL consider the intersection of race and ethnicity, age, education, and socioeconomic status in its definition, it is important that researchers and DA developers consider the role that each of these factors can play. For example, based on findings from this study, having pictures that represented their racial and ethnic background was important to the African American women in the study. Additionally, although we thought pictographs were simple to understand, we learned that was not the case for this population. Therefore, the challenge as we develop decision support tools for populations with predisposing risk factors for LHL is to consider both the overall message of what is being communicated as well as the ways in which they are affected by LHL.

Based on our qualitative interviews, it is essential that women be informed that mammography screening after age 75 years is a choice. We modified a mammography screening DA using the health literacy principles to account for populations with LHL. We now need to test it in practice with the goal of ensuring that all women know they have a choice regardless of literacy level.
